# Evidence of Cross-Reactive Immunity to 2009 Pandemic Influenza A Virus in Workers Seropositive to Swine H1N1 Influenza Viruses Circulating in Italy

**DOI:** 10.1371/journal.pone.0057576

**Published:** 2013-02-28

**Authors:** Maria A. De Marco, Stefano Porru, Paolo Cordioli, Bruno M. Cesana, Ana Moreno, Laura Calzoletti, Lebana Bonfanti, Arianna Boni, Antonio Scotto Di Carlo, Cecilia Arici, Angela Carta, Maria R. Castrucci, Isabella Donatelli, Paola Tomao, Vittoria M. Peri, Livia Di Trani, Nicoletta Vonesch

**Affiliations:** 1 Department of Infectious, Parasitic and Immune-Mediated Diseases, Istituto Superiore di Sanità, Rome, Italy; 2 Institute for Environmental Protection and Research (ISPRA), Ozzano Emilia, Bologna, Italy; 3 Department of Experimental and Applied Medicine, University of Brescia, Brescia, Italy; 4 Istituto Zooprofilattico Sperimentale della Lombardia e dell’Emilia Romagna, Brescia, Italy; 5 Department of Biomedical Sciences and Biotechnologies, University of Brescia, Brescia, Italy; 6 Istituto Zooprofilattico Sperimentale delle Venezie, Legnaro, Padua, Italy; 7 Department of Veterinary Public Health and Food Safety, Istituto Superiore di Sanità, Rome, Italy; 8 Department of Occupational Medicine, Italian Workers' Compensation Authority-Research Area (INAIL), Monteporzio Catone, Rome, Italy; Public Health Agency of Canada, Canada

## Abstract

**Background:**

Pigs play a key epidemiologic role in the ecology of influenza A viruses (IAVs) emerging from animal hosts and transmitted to humans. Between 2008 and 2010, we investigated the health risk of occupational exposure to swine influenza viruses (SIVs) in Italy, during the emergence and spread of the 2009 H1N1 pandemic (H1N1pdm) virus.

**Methodology/Principal Findings:**

Serum samples from 123 swine workers (SWs) and 379 control subjects (Cs), not exposed to pig herds, were tested by haemagglutination inhibition (HI) assay against selected SIVs belonging to H1N1 (swH1N1), H1N2 (swH1N2) and H3N2 (swH3N2) subtypes circulating in the study area. Potential cross-reactivity between swine and human IAVs was evaluated by testing sera against recent, pandemic and seasonal, human influenza viruses (H1N1 and H3N2 antigenic subtypes). Samples tested against swH1N1 and H1N1pdm viruses were categorized into sera collected before (n. 84 SWs; n. 234 Cs) and after (n. 39 SWs; n. 145 Cs) the pandemic peak. HI-antibody titers ≥10 were considered positive. In both pre-pandemic and post-pandemic peak subperiods, SWs showed significantly higher swH1N1 seroprevalences when compared with Cs (52.4% vs. 4.7% and 59% vs. 9.7%, respectively). Comparable HI results were obtained against H1N1pdm antigen (58.3% vs. 7.7% and 59% vs. 31.7%, respectively). No differences were found between HI seroreactivity detected in SWs and Cs against swH1N2 (33.3% vs. 40.4%) and swH3N2 (51.2 vs. 55.4%) viruses. These findings indicate the occurrence of swH1N1 transmission from pigs to Italian SWs.

**Conclusion/Significance:**

A significant increase of H1N1pdm seroprevalences occurred in the post-pandemic peak subperiod in the Cs (p<0.001) whereas SWs showed no differences between the two subperiods, suggesting a possible occurrence of cross-protective immunity related to previous swH1N1 infections. These data underline the importance of risk assessment and occupational health surveillance activities aimed at early detection and control of SIVs with pandemic potential in humans.

## Introduction

Viral exchange between human and animal populations is a crucial element in the origin of influenza pandemic strains. As in the pandemics of 1918, 1957 and 1968, caused by H1N1, H2N2 and H3N2 influenza A virus (IAV) antigenic subtypes, respectively, in the early third millennium the animal reservoir has played a key role in the origin and emergence of the 2009 H1N1 pandemic (H1N1pdm) virus, turned out to be a quadruple reassortant containing IAV genes from pigs, birds and humans [1]. Among animal species, pigs play an important role in influenza interspecies transmission, associated with viral gene reassortment, possibly occurring in pig tracheal epithelial cells during simultaneous infection of both avian and mammalian IAVs [2]. In addition, it is well known the ability of pigs to serve as intermediate hosts for the adaptation of avian viruses to mammals, including humans [3].

Swine influenza monitoring programs have been in place in Italy since 1990 s [4] and currently three prevalent antigenic subtypes, belonging to the Eurasian avian-like H1N1 and human-like H3N2 and H1N2 lineages, are circulating in Italian pigs [5]–[6]. The predominant swine H1N1 viruses have an entirely avian genome, and emerged in European pigs in 1979 [7]. The H3N2 swine viruses present in Europe since 1984 are human-avian reassortants, with avian-derived internal genes and haemagglutinin (HA) and neuraminidase (NA) genes of human origin [8]. The H1N2 swine influenza viruses (SIVs) currently circulating in Europe have the HA and NA genes of human origin and derive from viruses that were first introduced in Great Britain in 1994, as a result of multiple reassortment events involving initially human H1N1 and H3N2 and later avian-like swine H1N1 viruses [9]; H1N2 SIVs circulating in Italy have acquired, by a further reassortment event, a different NA deriving from recent human H3N2 viruses [6].

Since 1958, occasional cases of swine influenza infections have been reported in subjects with symptomatic infection, with the first SIV isolation in humans dated back to 1974 [10]. Seroepidemiological surveys were also carried out on swine-exposed workers to identify seroprevalences of antibodies against SIVs and to evaluate the public and occupational health impact. In particular, indirect evidence of SIV transmission to humans was found in America [11]–[17], Europe [18]–[20] and Asia [21].

The present study is part of a wide occupational medicine survey conducted from 2008 to 2010 in a densely pig-populated area of Northern Italy, and aimed to biologic risk assessment and to occupational health and safety appraisals, by means of worksites inspections, administration of specific questionnaires and seroprevalence surveys.

To assess the health risk of occupational exposure to swine influenza, sera from swine workers and controls were tested for the detection of antibodies against H1N1, H3N2 and H1N2 SIVs, circulating in the farms under study and representative of the above mentioned lineages [22]–[23]. To evaluate the antigenic cross-reactivity between HA proteins of SIVs and co-circulating human influenza A viruses, serum samples were also tested against seasonal H1N1 and H3N2 subtypes, and the H1N1pdm virus, which arose and spread in the human population [24] during the present study. Analysis of H1N1pdm antibody seroprevalences before and after the pandemic peak in Italy was also tailored to testing the hypothesis of the occurrence of cross-reactive immunity to the H1N1pdm virus in swine-exposed workers.

Our findings provide serologic evidence of swH1N1 transmission from pigs to Italian swine workers, and suggest that anti-swH1N1 antibodies have induced cross-protective immunity against the H1N1pdm virus.

## Materials and Methods

### Study Population

Between 15 December 2008 and 3 October 2010, sera were collected from 123 swine workers (SWs), operating in 23 farms of Lombardia Region, Northern Italy, that accounts for 51% of Italian pig population [25], and from 379 control subjects (Cs) employees in public and private companies of the same study area. SWs recruited represented 39.3% of individuals employed in the 23 farms under study. The time range of sera collection included the onset of the 2009 H1N1 pandemic, reaching its peak in Italy in the week 45 (2–8 November 2009) [26].

Socio-demographic and personal data, occupational history, history of influenza vaccination, individual and environmental biological risk factors, including exposure to pigs, were collected by an occupational health trained physician, through direct interview, by means of standardized questionnaires and workplace inspections. According to the Italian law, no ethical approval was needed, because the study was conducted as part of the occupational health surveillance program. A written informed consent was obtained from all participants.

### Laboratory Procedures

#### Antigenic characterisation of viruses

Swine influenza antigens were preliminarily selected among isolates from pigs reared in the 23 farms under study. Eighteen SIVs, including one H1N2, seven H1N1, ten H3N2 virus strains associated to outbreaks of acute respiratory disease occurring from 2006 to 2008, were characterized by cross-haemagglutination inhibition (cross-HI) test using three selected reference hyperimmune chicken sera, raised against co-circulating H1N1, H1N2, H3N2 antigenic subtypes of SIVs. Serologic cross-reactivity between swine and recent human influenza viral strains (seasonal H1N1 and H3N2, H1N1pdm) was assessed through cross-testing of reference antisera, according to current scientific findings [13], [15], [19]–[20].

#### Analysis of human sera

Receptor destroying enzyme (RDE) (Sigma, St. Louis, MO) treated sera were examined by a standardized haemagglutination inhibition (HI) method [27] using four haemagglutinating units (HAUs) of virus and 0.5% turkey red blood cells. HI titers were expressed as the reciprocal of the highest serum dilution inhibiting four HAUs of antigen and sera with HI titers ≥10 were considered positive for influenza antibodies.

Collected sera were assayed by the HI test against six strains of recently circulating swine and human influenza A viruses: A/Sw/Italy/44795/08 (swH1N1), A/Sw/Italy/114347–1/06 (swH1N2), A/Sw/Italy/32242/06 (swH3N2), A/Brisbane/59/07 (huH1N1), A/Uruguay/716/07 (A/Brisbane/10/07-like) (huH3N2), A/California/7/2009 (H1N1pdm). SIV strains, grown in embryonated chicken eggs, were representative of the Eurasian avian-like H1N1 and human-like H1N2 and H3N2 lineages, as shown by previous phylogenetic analyses of Italian isolates [22]–[23]. Vaccinal subunits were chosen as human influenza antigens since preliminary HI assays of human sera tested against both whole and purified antigens showed higher sensitivity associated with the purified ones (data not shown). Human influenza antigens were used to evaluate: i) the HI serologic cross-reactivity between SIVs belonging to human-like H1N2 and H3N2 lineages, and co-circulating seasonal strains (H1N1 and H3N2 subtypes, respectively); ii) the HI serologic cross-reactivity between Eurasian avian-like H1N1 swine influenza viruses and the H1N1pdm virus.

### Statistical Analysis

HI antibody titers and selected data from questionnaire (age, sex, vaccination history, exposure to pigs) of 502 participants were combined into a database, using SPSS v. 19 statistical software.

To assess the zoonotic potential of H1N1, H1N2 and H3N2 SIV infections, seroprevalence rates (SPRs) detected in SWs and Cs were compared. Levels of seropositivity to swH1N1 and H1N1pdm viruses detected in SWs and Cs before and after the pandemic peak were also compared, referring to conventional cut-off date of 1 November 2009, that was chosen taking into account the Italian epidemiological context [26]. HI SPRs were compared referring to three cutoff levels, ranging from 10 (minimum level of detection) to 40 (protective antibody titer) [28].

In geometric mean titer calculation, a titer less than 10 was assigned a value of 5.

Pearson’s chi-square or Fisher’s exact tests and bivariate logistic regression analysis were used as appropriate. When relevant, 95% confidence intervals (CI) were computed. P values <0.05 were considered as statistically significant.

## Results

### Study Population

Study and control subjects were categorized by age, sex and vaccination history as shown in Table 1. Mean age of the 123 SWs was 45.05 years (range 17–75 years), and 95.1% were male. Mean age of the Cs was 39.75 (range 20–61), and 76.5% were male. The Chi-square test showed significant differences in age and sex distribution. Cs showed a greater proportion of females, more subjects under 40 years of age and fewer in the >50 years age class. Low vaccination coverage was detected both in the control population and in risk group, without significant differences.

**Table 1:** Characteristics of the swine worker and control groups.

Variable	Subcategory	Study period						
		Pre-pandemic peak∧		Post-pandemic peak∧			Total	
		SWs, n (%)	Cs, n (%)	SWs, n (%)	Cs, n (%)	SWs, n (%)		Cs, n (%)
		84 (68.3)	234 (61.7)	39 (31.7)	145 (38.3)	123 (100)		379 (100)
Age	≤40 years†	24 (28.6)	111 (47.4)	16 (41.0)	85 (58.6)	40 (32.5)		196 (51.7)
	41–50 years	33 (39.3)	77 (32.9)	12 (30.8)	38 (26.2)	45 (36.6)		115 (30.3)
	>50 years†	27 (32.1)	46 (19.7)	11 (28.2)	22 (15.2)	38 (30.9)		68 (17.9)
Sex†	Male	79 (94.0)	161 (68.8)	38 (97.4)	129 (89.0)	117 (95.1)		290 (76.5)
	Female	5 (6.0)	73 (31.2)	1 (2.6)	16 (11.0)	6 (4.9)		89 (23.5)
Seasonal	Yes	16 (19.0)	61 (26.1)	7 (17.9)	27 (18.6)	23 (18.7)		88 (23.2)
vaccine*	No	68 (81.0)	173 (73.9)	32 (82.1)	118 (81.4)	100 (81.3)		291 (76.8)

* Seasonal vaccine was defined as any previous vaccination against seasonal influenza.

† p<0.05. The calculated p-value is referred to total numbers of swine workers (SWs) and controls (Cs).

∧ Pandemic peak cut-off date 1 November 2009.

### Antigenic Characterisation of Viruses

Serologic characterization by cross-HI test has shown a high degree of antigenic homology within all the examined H1N1 (Table 2) and H3N2 (Table 3) SIVs. A/Sw/Italy/44795/08 (swH1N1), A/Sw/Italy/114347–1/06 (swH1N2), A/Sw/Italy/32242/06 (swH3N2), were finally selected.

**Table 2:** Swine and human influenza viruses: HI antigenic characterization of H1 strains.

		HI titer of serum against:			
Virus origin	Virus strain	A/Sw/It/114347–1/06 (H1N2)*	A/Sw/It/125746/05 (H1N1)*	A/Brisbane/59/07 (H1N1)*	A/California/7/09 (H1N1)**
Swine	A/Sw/It/114347-1/06, H1N2 ∧	**80**	<10	<10	<10
Swine	A/Sw/It/125746/05, H1N1	<10	**1280**	10	80
	A/Sw/It/66732/2/06, H1N1∧	<10	1280	<10	320
	A/Sw/It/87491/06, H1N1∧	<10	1280	<10	160
	A/Sw/It/45894/2/07, H1N1∧	<10	640	<10	160
	A/Sw/It/68030/07, H1N1∧	<10	640	<10	160
	A/Sw/It/232868/07, H1N1∧	<10	1280	<10	320
	A/Sw/It/207871/08, H1N1∧	<10	640	<10	160
	A/Sw/It/44795/08, H1N1 ∧	<10	640	<10	160
Human	A/Brisbane/59/07, H1N1	10	20	**1280**	<10
	A/California/7/09, H1N1	<10	160	<10	**1280**

HI, Haemagglutination inhibition.

* Chicken hyperimmune antiserum.

** Ferret post-infection antiserum.

Virus strains used to test swine workers’ and controls’ sera are underlined.

∧ SIVs strains isolated from pigs, in the farms under study.

**Table 3:** Swine and human influenza viruses: HI antigenic characterization of H3 strains.

		HI titer of serum against:	
Virus origin	Virus strain	A/Sw/It/79604/06 (H3N2)*	A/Brisbane/10/07 (H3N2)*
Swine	A/Sw/It/79604/06, H3N2	**2560**	<10
	A/Sw/It/32242/06, H3N2 ∧	1280	<10
	A/Sw/It/20319/06, H3N2∧	1280	<10
	A/Sw/It/186423/07, H3N2∧	640	<10
	A/Sw/It/206453/07, H3N2∧	1280	<10
	A/Sw/It/319388/07, H3N2∧	1280	<10
	A/Sw/It/209720/08, H3N2∧	1280	<10
	A/Sw/It/136775/08, H3N2∧	1280	<10
	A/Sw/It/120014/08, H3N2∧	1280	<10
	A/Sw/It/117304/08, H3N2∧	640	<10
	A/Sw/It/235509/08, H3N2∧	640	<10
Human	A/Uruguay/716/07, H3N2	<10	**2560**

HI, Haemagglutination inhibition.

* Chicken hyperimmune antiserum.

Virus strains used to test swine workers’ and controls’ sera are underlined.

∧ SIVs strains isolated from pigs, in the farms under study.

Swine H1N1 antiserum showed cross-reactivity against human H1N1pdm virus, with HI titer of 160; comparable results were also obtained by testing human H1N1pdm antiserum against swH1N1 viruses. Low cross-reaction was detected with swine H1N2 and swine H1N1 antisera against human H1N1 seasonal virus (HI titers of 10 and 20 respectively), whereas antiserum against human H1N1 seasonal virus did not cross-react with swine H1N1 and H1N2 viruses under study (Table 2).

Finally, no cross-reactivity was detected between recent swine and human H3N2 influenza viruses (Table 3).

### Serological Results of the Whole Study Period

To investigate the health risk of occupational exposure to SIVs, HI antibody reactivities in sera of SWs and Cs were compared as shown in Table 4.

**Table 4:** HI antibody reactivity of SWs and Cs sera against swine and human influenza viruses during the whole study period (Italy, 2008–2010)§.

Virus	Cutoff value	SWs, N = 123	Cs, N = 379	Unadjusted OR	Adjusted OR∧
		n (%)	n (%)	(95% CI)	(95% CI)
swH1N1	≥10	67 (54.5)	25 (6.6)	16.9 (9.9–29.0)***	17.2 (2.3–0.2)***
	≥20	39 (31.7)	13 (3.4)	13.1 (6.7–25.6)***	12.3 (6.3–25.0)***
	≥40	14 (11.4)	7 (1.8)	6.8 (2.7–17.3)***	6.6 (2.5–17.4)***
huH1N1pdm	≥10	72 (58.5)	64 (16.9)	6.9 (4.4–10.9)***	7.3 (4.6–12.6)***
	≥20	62 (50.4)	45 (11.9)	7.5 (4.7–12.1)***	7.6 (4.7–12.4)***
	≥40	39 (31.7)	32 (8.4)	5.0 (3.0–8.5)***	5.3 (3.1–9.2)***
swH1N2	≥10	41 (33.3)	153 (40.4)	0.7 (0.5–1.1)	0.7 (0.5–1.1)
	≥20	30 (24.4)	93 (24.5)	1.0 (0.6–1.6)	1.0 (0.6–1.7)
	≥40	19 (15.4)	44 (11.6)	1.4 (0.8–2.5)	1.5 (0.8–2.8)
huH1N1	≥10	58 (47.2)	228 (60.2)	0.6 (0.4–0.9)**	0.6 (0.4–0.9)**
	≥20	45 (36.6)	193 (50.9)	0.6 (0.4–0.8)**	0.6 (0.4–0.9)**
	≥40	35 (28.5)	150 (39.6)	0.8 (0.4 –1.4)	nc
swH3N2	≥10	63 (51.2)	210 (55.4)	0.8 (0.6–1.3)	0.8 (0.5–1.3)
	≥20	49 (39.8)	161 (42.5)	0.9 (0.6–1.4)	1 (0.6–1.4)
	≥40	34 (27.6)	110 (29.0)	0.9 (0.6–1.5)	0.9 (0.6–1.5)
huH3N2	≥10	51 (41.5)	218 (57.5)	0.5 (0.3–0.8)**	0.8 (0.6–1.3)**
	≥20	43 (35.0)	188 (49.6)	0.5 (0.4–0.8)**	0.9 (0.6–1.4)**
	≥40	35 (28.5)	147 (38.8)	0.6 (0.4–0.1)*	1 (0.6–1.6)*

§ Values are no. persons with antibodies.

∧ Data adjusted by age in binary logistic regression.

HI, Haemagglutination inhibition.

SWs, swine workers; Cs, controls.

OR, odds ratio; CI, confidence interval; nc, not calculated.

swH1N1, A/Swine/Italy/44795/08; huH1N1pdm, A/California/7/09;

swH1N2, A/Swine/Italy/114347–1/06; huH1N1, A/Brisbane/59/07;

swH3N2, A/Swine/Italy/32242/06; huH3N2, A/Uruguay/716/07.

Statistically significant values: *, p<0.05; **, p<0.01; ***, p<0.001.

Taking into account the antigenic cross-reactivity (Table 2) and the HA genetic origin of viral strains [29], results of paired swine and human viruses were analyzed as follows:

#### Swine avian-like H1N1 and H1N1pdm influenza viruses

Comparison between swH1N1seroprevalences showed significant higher values in SWs compared with Cs at cutoff titer of ≥10, ≥20, ≥40. Comparable seroprevalence results were obtained using the H1N1pdm antigen in HI assay, showing that antibody responses against swH1N1 virus and H1N1pdm virus correlated with each other as detected by cross HI-test (Table 2).

Bivariate logistic regressions showed significant difference between HI seroprevalence rates (SPRs) detected in SWs and Cs, at all cut-off levels, for swH1N1 and H1N1pdm viruses, with evidence of an age effect. Adjusted odds ratios have been calculated to take into account the unbalanced age distribution between swine workers and controls (age strata as ≤40, 41–50 and >50 were considered).

#### Swine human-like H1N2 and seasonal H1N1 influenza viruses

Bivariate logistic regressions showed no differences between HI seroreactivity detected in SWs and Cs against the swH1N2 virus, at each cutoff value. SPRs for huH1N1 virus were higher in Cs than in SWs, with significant differences only at cutoff titers of 10 and 20 levels.

#### Swine human-like H3N2 and seasonal H3N2 influenza viruses

No differences were found between HI seroreactivity detected in SWs and Cs against the swH3N2 virus, at each cutoff value. SPRs for huH3N2 virus were significantly higher in Cs than in SWs at all cutoff titers.

### Pre- and Post-pandemic Peak Periods: Comparison of Serological Results between and Within SWs and Cs

**Figure 1:**
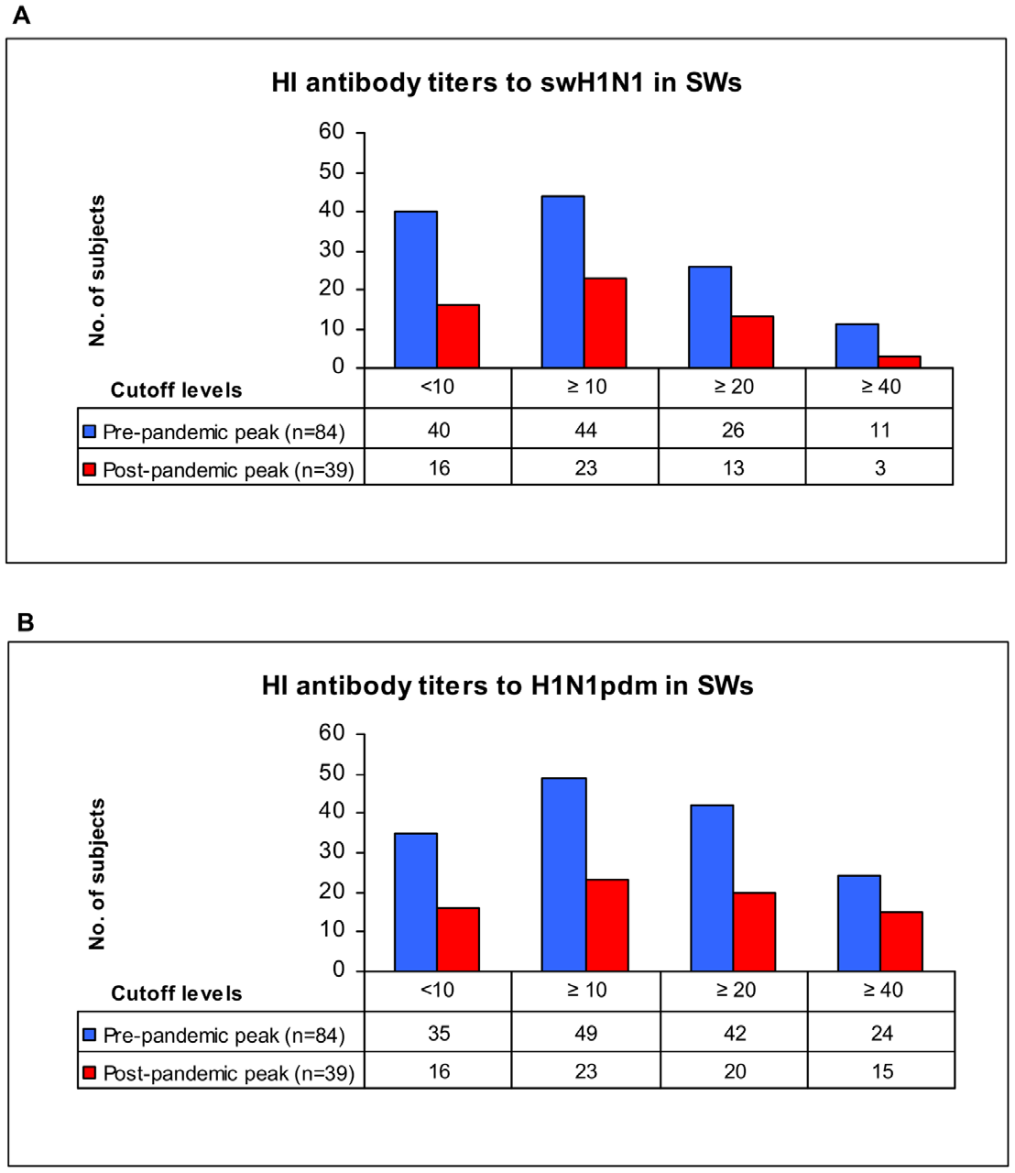
HI antibody reactivity against swH1N1 and H1N1pdm viruses in SWs (Italy, 2008–2010). Serum samples were collected from swine workers (SWs) in the pre-pandemic peak (15 December 2008–1 November 2009) and post-pandemic peak (2 November 2009–3 October 2010) periods and tested by haemagglutination inhibition (HI) assay. Individual HI results referred to the three cutoff levels (≥10, ≥20 and ≥40) chosen for the statistical analysis of data, and ranging from 10 (minimum level of detection) to 40 (protective antibody titer). No significant difference in seroprevalence rate (SPR) was found in SWs sera tested by HI assay against swH1N1 (Figure 1A) and H1N1pdm (Figure 1B) viruses (viruses details and SPRs are shown in Table 5).

**Figure 2:**
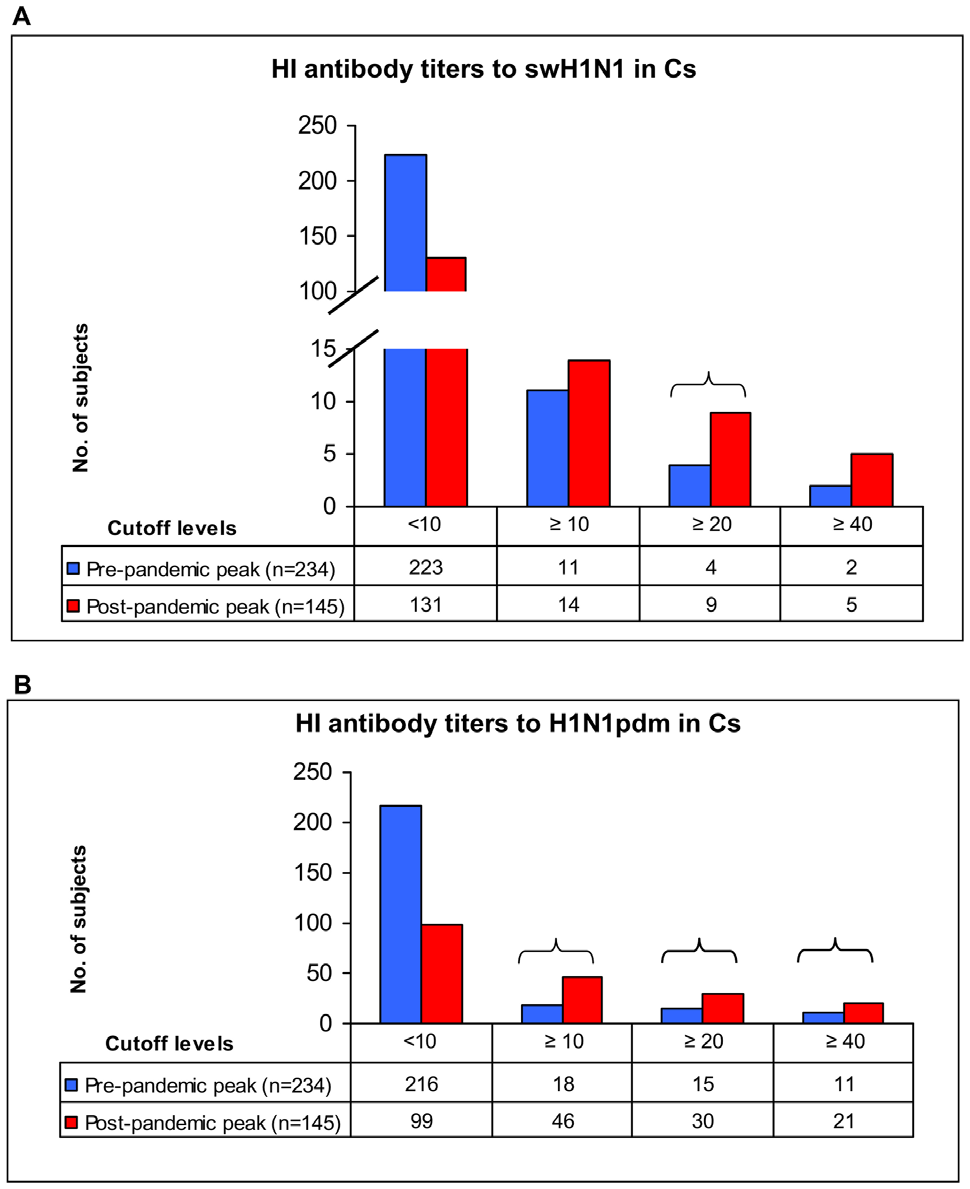
HI antibody reactivity against swH1N1 and H1N1pdm viruses in Cs (Italy, 2008–2010). Serum samples were collected from control subjects (Cs) in the pre-pandemic peak (15 December 2008–1 November 2009) and post-pandemic peak (2 November 2009–3 October 2010) periods and tested by haemagglutination inhibition (HI) assay. Individual HI results referred to the three cutoff levels (≥10, ≥20 and ≥40) chosen for the statistical analysis of data, and ranging from 10 (minimum level of detection) to 40 (protective antibody titer). A significant increase in seroprevalence rate (SPR) was found against swH1N1 at cutoff ≥20 (Figure 2A), whereas H1N1pdm SPRs (Figure 2B) were significantly higher in the post-pandemic peak period, at all cutoff titer levels (viruses details and SPRs are shown in Table 5). SPR significant difference (p<0.05).

Taking into account the conventional 1 November 2009 cutoff date (pre- and post- pandemic peak), seroprevalences to swH1N1 and H1N1pdm viruses were compared, as shown in Table 5, Figures 1 and Figure 2. Binary logistic regression showed higher SPRs in SWs in both pre- and post-pandemic period (Table 5), at all cutoff levels with the exception of cutoff ≥40 for swH1N1 in the post-pandemic period, where very low cell frequency was observed.

**Table 5:** SWs and Cs sera collected before and after the 2009 pandemic influenza peak: HI antibody reactivity against swH1N1 and H1N1pdm viruses§.

Virus	Cutoff value	Pre-pandemic peak period			Post-pandemic peak period		
		(15 Dec. 2008–1 Nov. 2009)			(2 Nov. 2009–3 Oct. 2010)		
		SWs	Cs		SWs	Cs	
		N = 84	N = 234	OR	N = 39	N = 145	OR
		n (%)	n (%)	(95% CI)	n (%)	n (%)	(95% CI)
swH1N1	≥10	44	11	22.2	23	14	13.6
		(52.4)	(4.7)	(10.6–46.6)***	(59.0)	(9.7)	(5.8–31.5)***
	≥20	26	4	25.7	13	9	7.6
		(31.0)	(1.7)	(8.6–76.4)***	(33.3)	(6.2)	(2.9–19.7)***
	≥40	11	2	17.4	3	5	2.3
		(13.1)	(0.9)	(3.8–80.3)***	(7.7)	(3.4)	(0.5–10.3)
huH1N1pdm	≥10	49	18	16.7	23	46	3.1
		(58.3)	(7.7)	(8.7–32)***	(59)	(31.7)	(1.5–6.5)**
	≥20	42	15	14.5	20	30	4.1
		(50.0)	(6.4)	(7.4–28.6)***	(51.3)	(20.7)	(1.9–8.6)***
	≥40	24	11	8.1	15	21	3.7
		(28.6)	(4.7)	(3.7–17.4)***	(38.5)	(14.5)	(1.6–8.2)**

§ Values are no. persons with antibodies.

SWs, swine workers; Cs, controls; OR, odds ratio; CI, confidence interval.

swH1N1,/Swine/Italy/44795/08; huH1N1pdm, A/California/7/09.

Statistically significant values: *, p<0.05; **, p<0.01; ***, p<0.001.

Comparing pre- and post- pandemic peak HI results, no significant difference in SPRs was found in SWs sera tested against H1N1pdm (Figure 1B) and SwH1N1 (Figure 1A) viruses. Conversely, H1N1pdm SPRs were significantly higher in Cs sera collected during the post- pandemic peak period, at all cutoff titer levels (Figure 2B); a significant increase in SPRs was also found to SwH1N1 at cutoff ≥20 (Figure 2A).

The overall proportion of SWs with antibody levels ≥40 against H1N1pdm was 31.7% (Table 4), ranging from 28.6% in the pre-pandemic peak period to 38.5% in the post-pandemic peak one (Table 5). In logistic regression models the seropositivity of SWs to swH1N1 (HI titers ≥20 and ≥40) was associated with antibody levels ≥40 against H1N1pdm, supporting the existence of cross-reactivity between these viruses; age did not appear to be an explanatory variable.

Geometric mean titers (GMTs) of antibodies to H1N1pdm virus detected in SWs in the pre-pandemic and post-pandemic peak periods were 14.86 *vs.* 16.45, respectively; in the same periods, GMTs of H1N1pdm antibodies in Cs were 5.93 vs. 9.22.

## Discussion

Serosurveys represent an useful tool to highlight the zoonotic risk posed by swine influenza, allowing a better quantification of SIV associated human infections when compared to surveillance systems based on virological diagnosis of influenza-like illness [10]. Seroprevalence studies provided indirect evidence of SIV transmission to swine-exposed workers in North America [11]–[17], Europe [18]–[20] and Thailand [21].

The Italian pig farming developed in the 1960s in the Po valley; in 2007 the Italian pig population was more than 9 million heads, mainly concentrated in the four Northern regions of Lombardia, Emilia-Romagna, Piemonte and Veneto [30].

The present observational cross-sectional study of occupational medicine, conducted in the Lombardia Region between 2008 and 2010, was planned to assess the risk of occupational exposure to swine influenza, by testing human sera against SIVs representative of the Eurasian avian-like H1N1 and human-like H3N2 and H1N2 lineages [22]–[23].

It should be noted that concurrent veterinary SIV monitoring programs in the pig population [4] allowed us to: i) identify, in a densely populated pig area, SIV infected farms; ii) choose, by antigenic characterisation (Tables 2 and Table3), SIV strains to be used in the present serosurvey; iii) test SWs sera against selected SIV strains to which they were exposed.

Table 1 provides some demographic and individual characteristics of worker groups exposed and unexposed to SIVs. Only 18.7% of SWs and 23.2% of Cs were vaccinated against seasonal influenza (Table 1) showing vaccination coverages lower than values recently reported in Luxembourg [19] and Germany [20].

Study results (ORs reported in Table 4 and Table 5) show that SWs are at higher risk than Cs for swH1N1 virus infection, caused by the prevalent SIV antigenic subtype, which is endemic in Italian pig farms [23], thus showing much greater odds than did Cs of being seropositive against both the swH1N1 and H1N1pdm viruses (Table 4). Our results are consistent with previous serologic studies conducted in Europe, showing that subjects occupationally exposed to swine had more anti-swH1N1 antibodies than controls [19]–[20]. Similar studies, in North America and Thailand, showed antibody seroprevalences against classical swine H1N1 viruses [11]–[16], [21].

As detected by cross HI-test (Table 2) seroprevalence results confirmed that antibody responses against swH1N1 and H1N1pdm viruses correlated with each other, despite the wide antigenic and genetic distance between the H1 haemagglutinin of the European avian-like H1N1 SIV and H1N1pdm virus [29]. HI antibodies showing “unexpected” cross-reactivities against H1N1pdm strains, have also been detected in swine workers and pigs infected and/or vaccinated, respectively, with European avian-like H1N1 SIVs [19], [31].

Notwithstanding the HA gene human origin of the swH1N2 virus used in this study, the antigenic characterization (Table 2) showed very low cross-reaction of swH1N2 antiserum against human H1N1 seasonal virus (HI titers of 10). Similarly, although HA gene of swH3N2 derived from human influenza viruses, no cross-reactivity was detected between antiserum raised against this SIV strain and the seasonal H3N2 virus (Table 3). Characterization results can be explained by the much slower evolution rate of influenza A viruses circulating in pigs, when compared to that of the human counterparts [29], [31]. However, in the present serosurvey comparable HI seroreactivities were detected at each HI cutoff value (Table 4) in SWs and Cs against both swH1N2 and swH3N2 viruses; these data can be explained with the lifetime exposure of both study groups to human seasonal influenza viruses, resulting in a broad heterosubtypic immunity [32] and subsequent complexity to discriminate specific antibody responses against swine and human viruses. Notably, as also described by López -Robles et al. in Northwestern Mexico [17], our study showed lower percentages of seropositives for huH1N1 and huH3N2 viruses in SWs compared to the Cs (Table 4).

The present occupational study is unique in that its time frame included the complete periods of onset and global spread of the H1N1 2009 influenza pandemic [26], [33], giving us the opportunity to compare the immune response in study groups (SWs and Cs) before and after the pandemic peak.

SWs were at higher risk than Cs for swH1N1 infection in the whole study period, in the pre- and in the post-pandemic periods. Across the cutoff levels, ORs of having antibodies against swH1N1 were 6.6 to 17.2 greater for SWs than for Cs in the whole period, 17.4 to 22.2 in the pre-pandemic period and 2.3 to 13.6 in the post-pandemic period. However, ORs decreased after the pandemic peak (cutoff ≥10, 22.2 *vs.* 13.6; cutoff ≥20, 25.7 *vs.* 7.6; cutoff ≥40, 17.4 *vs.* 2.3) as a result of a significant increase of cross-reactive antibody response in Cs, due to probable H1N1pdm infections (Table 5 and Figure 2A). Consistent with the above mentioned cross-reactivity (swine and pandemic H1N1 viruses), the same trend is observed for H1N1pdm (cutoff ≥10, 16.7 *vs.* 3.1; cutoff ≥20, 14.5 *vs.* 4.1; cutoff ≥40, 8.1 *vs.* 3.7); post-pandemic lower ORs values account for a significant increase of control subjects with antibodies against H1N1pdm (Table 5 and Figure 2B).

The opportunity to analyze the antibody results of sera collected during the pandemic period, allowed us to postulate that anti-swH1N1 antibodies have induced cross-reactive immunity in SWs against the H1N1pdm virus. In fact, the significant increase of H1N1pdm seroprevalences occurred in the post-pandemic peak period in the Cs group (Table 5 and Figure 2B), compared to the lack of significant differences observed in SWs (Table 5 and Figure 1B), suggests the occurrence of cross-protective immunity to H1N1pdm virus in Italian SWs, previously infected by swH1N1viruses.

The overall proportion of SWs with antibody levels ≥40 against H1N1pdm was 31.7% (Table 4), ranging from 28.6% in the pre-pandemic peak period to 38.5% in the post-pandemic peak one (Table 5). All the above values could have contributed to protection against the H1N1pdm virus.

GMTs to H1N1pdm virus we detected in Cs in the pre-pandemic and post-pandemic peak periods (5.93 *vs*. 9.22, respectively) are in agreement with GMT values previously reported in other European countries [34].

Our data of seroprevalence and risk analysis may have public health impact, providing elements useful for the prevention and control of influenza A viruses with pandemic potential. Such actions may include both direct prophylaxis activities, designed to prevent SIV infection in occupationally exposed workers, and vaccination measures, aimed to counteract co-infections of SWs with influenza A viruses of human and animal origin, underlying the possible emergence of new pandemic strains by reassortment events [35]–[36]. Moreover, we found that only a limited number of SWs were vaccinated against human influenza (Table 1), emphasising the importance of including swine workers in seasonal and pandemic influenza prevention planning.

In addition, our findings enhance the understanding of cross-reactivity immunity elicited by swine and human influenza virus infections, recently analyzed in humans [37] and animal models [31], [38]–[39]. In the present study we postulated that prior infection with swH1N1 virus can provide cross-protective humoral immunity to H1N1pdm virus. However, it is important to consider that pre-existing T cell-mediated heterologous immunity could also have contributed to protect SWs against H1N1pdm virus infection [40].

Since H1N1pdm pandemic became a matter of fact during the survey period, the present study was not specifically designed to test the presence of humoral immunity against H1N1pdm in humans previously exposed to swH1N1 viruses; sampling procedures were neither consequently tailored for this purposes, nor balanced for temporal comparison between pre- and post-pandemic peak periods. However despite all these limitations, to our knowledge, this study is unique in documenting exposure of SWs and control population to SIVs and human influenza viruses at the onset and during the spread of the 2009 influenza pandemic.

In summary, our findings provide new insights into the human immunity to swine influenza viruses. We also confirm that swine workers are at significant risk to become infected by SIVs, thus underlying the crucial role of an integrated occupational medicine and veterinary surveillance aimed to assess the health risk represented by zoonotic viruses.
